# Early Efficacy and Exploratory Biomarker Data of Bel-sar in Patients with Intermediate-risk and High-risk Non–muscle-invasive Bladder Cancer

**DOI:** 10.1016/j.euros.2026.05.017

**Published:** 2026-06-13

**Authors:** Jennifer Linehan, Piyush K. Agarwal, Xianne Penny, Max Kates, Joseph Jacob, Seth P. Lerner, Rhonda C. Kines, John T. Schiller, Elisabet de los Pinos, Neal D. Shore, Jill Hopkins, Joseph McQuaid, Sabine D. Brookman-May

**Affiliations:** a John Wayne Cancer Institute, Santa Monica, USA; b University of Chicago, Chicago, USA; c Aura Biosciences, Boston, USA; d Johns Hopkins School of Medicine, Baltimore, USA; e SUNY Upstate Medical University, Syracuse, USA; f Baylor College of Medicine, Houston, USA; g Laboratory of Cellular Oncology, NCI, NIH, Bethesda, USA; h START Carolinas/Carolina Urologic Research Center, Myrtle Beach, USA; i University of Munich, LMU, Munich, Germany

**Keywords:** Belzupacap sarotalocan, Immunogenic cell death, Non–muscle-invasive bladder cancer, Photoimmunotherapy, Tertiary lymphoid structures, Tumor microenvironment, Urothelial carcinoma, Virus-like drug conjugate

## Abstract

**Background and objective:**

Despite available therapies, patients with non–muscle-invasive bladder cancer (NMIBC) are impacted by recurrence and progression risk. Bel-sar (belzupacap sarotalocan) is a first-in-class virus-like drug conjugate composed of a tumor-targeting virus-like particle linked to a photoactivatable dye, inducing proimmunogenic tumor cell death and antitumor immune response. We report clinical and exploratory immune biomarker data from a phase 1 study in intermediate-risk and high-risk NMIBC.

**Methods:**

Seventeen participants received focally injected bel-sar (100–200 µg) without (*n* = 5) or with (*n* = 12) light activation, followed by transurethral resection of bladder tumor (TURBT; 7–12 d later). Safety, feasibility, tolerability, and preliminary efficacy were assessed. Multiplex immunofluorescence was performed on paired tumor specimens from five responders receiving light-activated bel-sar.

**Key findings and limitations:**

Bel-sar was well tolerated, with only grade 1 adverse events, and no grade ≥2, serious, or dose-limiting toxicities. Among 10 efficacy-evaluable patients receiving light activation, four of five low-grade tumors achieved complete response. Responses were also observed in high-grade and untreated tumors, suggesting a urothelial field effect. Immune profiling demonstrated conversion of immune-cold or exhausted tumors into immunogenically primed tumors, with tertiary lymphoid structure formation, expansion of cytotoxic and memory CD4+ T cells, and marked natural killer cells and eosinophils recruitment. Limitations include the small sample size and short follow-up.

**Conclusions and clinical implications:**

Bel-sar demonstrated focal administration feasibility, a favorable safety profile, and encouraging preliminary efficacy in NMIBC, with robust immune activation in treated and untreated tumors. These findings support bel-sar’s continued development as a therapy combining local tumor eradication with durable immune surveillance.

## Take Home Message

Bel-sar demonstrated feasibility, safety, and early efficacy in non-muscle-invasive bladder cancer, with clinical efficacy and immune activation extending beyond treated lesions. Induction of tertiary lymphoid structures and immune memory supports its potential to achieve durable tumor control with reduced treatment burden.

ADVANCING PRACTICE **What does this study add?**

This study demonstrates that focal administration of bel-sar is feasible and well tolerated in patients with intermediate- and high-risk non**–**muscle-invasive bladder cancer. It provides early evidence of antitumor activity, including responses in nontreated lesions. Importantly, the study shows robust innate and adaptive immune activation, with induction of tertiary lymphoid structures and immune memory, supporting bel-sar’s dual cytotoxic and immune-mediated mechanism of action.

**Clinical Relevance**

Patients with Non–Muscle-Invasive Bladder Cancer continue to face high recurrence rates, treatment burden, and limited durable immune control despite current intravesical therapies. This study provides early clinical and translational evidence that bel-sar may offer a novel immune-ablative approach by combining focal tumor destruction with activation of both innate and adaptive antitumor immunity. The observed responses in treated and non-treated lesions, together with induction of tertiary lymphoid structures and immune memory, suggest the potential not only for local tumor eradication but also for durable urothelial immune surveillance. If confirmed in larger studies, bel-sar could represent a new treatment paradigm with reduced procedural burden and improved long-term disease control for patients with NMIBC. Associate Editor: M. Carmen Mir, M.D., PhD.

**Patient Summary**

This study tested a new treatment for early-stage bladder cancer consisting of a virus-like particle and a photoactivated dye that combines targeted tumor destruction with immune activation. The treatment was safe and showed early signs of efficacy, with tumor resolution and immune activation even in areas that were not directly treated. It may help reduce cancer recurrence with fewer treatments.

## 1 Introduction

Non–muscle-invasive bladder cancer (NMIBC) accounts for approximately 75% of newly diagnosed bladder cancers and represents a heterogeneous disease with variable recurrence and progression risks [1], [2]. Standard management includes transurethral resection of the bladder tumor (TURBT), followed by intravesical chemotherapy or Bacillus Calmette–Guérin (BCG). Although BCG remains the cornerstone of treatment, particularly for high-risk (HR) disease, both BCG and chemotherapy are limited by toxicity, treatment burden, and recurrence rates [3].

Several novel agents—including immune modulators, fibroblast growth factor receptor (FGFR) inhibitors, oncolytic agents, and new chemotherapy formulations—are under investigation; however, durable efficacy with favorable tolerability remains elusive [4]. Immunotherapies are of particular interest given their potential to elicit tumor-specific immune responses.

**Fig. 1:**
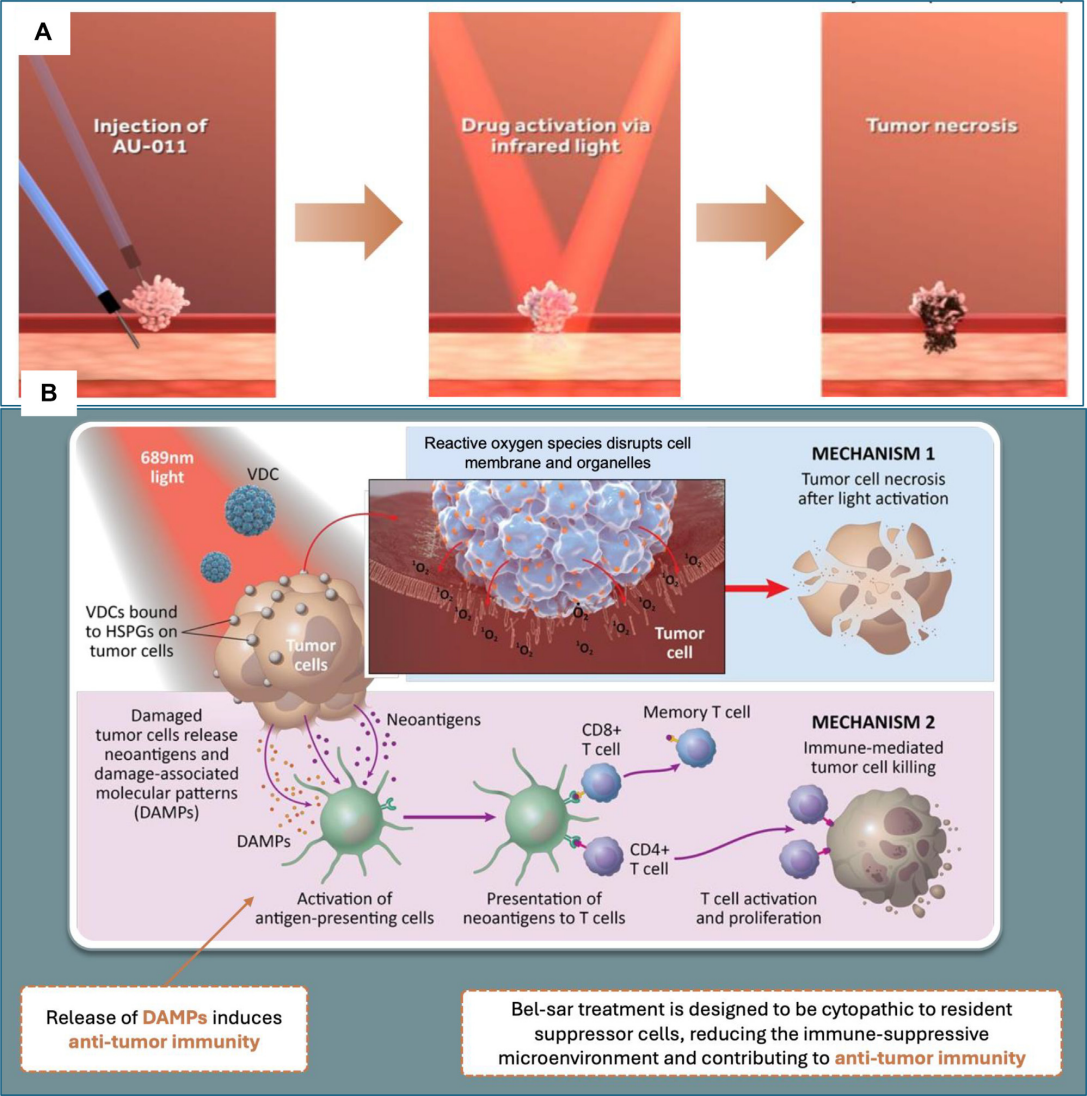
Schematic representation of bel-sar-mediated tumor ablation (A) and proposed mechanism of action of belzupacap sarotalocan (bel-sar, AU-011) (B). bel-sar = belzupacap sarotalocan; VDC = virus-like drug conjugate; HSPG = heparan sulfate proteoglycan; DAMPs = damage-associated molecular patterns. Bel-sar is a VDC composed of human papillomavirus–derived virus-like particles conjugated to a photosensitizer. Following intratumoral injection of bel-sar, VDC particles selectively bind to tumor cells via HSPGs expressed on the tumor cell surface. Subsequent activation with near-infrared light induces photochemical disruption of tumor cell membranes, resulting in necrotic tumor cell death. This process releases DAMPs and tumor antigens and may stimulate innate and adaptive antitumor immune responses, potentially affecting also untreated lesions.

Bel-sar (belzupacap sarotalocan) is a first-in-class, nonreplicating virus-like drug conjugate (VDC) composed of a tumor-targeting virus-like particle (VLP) linked to a photoactivatable phthalocyanine dye with a dual mechanism of action. After selective binding to tumor cells via heparan sulfate proteoglycans, subsequent near-infrared light activation (689 nm) induces reactive oxygen species-mediated tumor necrosis while sparing surrounding tissue (Fig. 1). This proimmunogenic cell death elicits T-cell-mediated and tumor-associated immune activation, coupling tumor ablation with immune priming [5], [6], [7].

Here, we report initial safety and efficacy in participants with intermediate-risk (IR) and HR NMIBC, along with exploratory immune biomarker analyses in selected responders, from the phase 1 AU-011-102 study, which included an initial safety cohort without light activation (*n* = 5) followed by window-of-opportunity cohorts prior to TURBT (*n* = 12).

## 2 Patients and methods

### 2.1 AU-011-102 Study Design

AU-011-102 (NCT05483868) is an open-label, multicenter study evaluating focal bel-sar in participants with IR and HR NMIBC, approved by the Sterling Central Institutional Review Board (Registration Number: IRB00001790) and conducted in accordance with the Declaration of Helsinki. Participants were enrolled consecutively between September 2022 and April 2025.

Baseline characteristics of the participants enrolled are summarized in Table 1. Participants had at least one visible papillary tumor amenable to cold-cup biopsy for histopathologic confirmation. Risk classification followed American Urological Association (AUA) criteria (2020), with efficacy analyses additionally performed using the International Bladder Cancer Group (IBCG) system (Table 2) [8], [9]. Primary study objectives included safety, feasibility, focal drug distribution, tumor necrosis, and preliminary efficacy, including tumor and immune responses.

**Table 1:** Clinicopathologic characteristics of 17 participants with NMIBC who received a single intratumoral injection of AU-011

**Characteristics**	**Study Cohort (*n*** = **17)**
Male Gender, *n* (%)	14 (82)
Age, median (Q1; Q3)	72 (63, 81)
Recurrent Disease at screening, *n* (%)	12 (71)
Multiple tumors at screening, *n* (%)	9 (53)
Maximum tumor diameter < 3 cm at screening, *n* (%)	13 (77)
Clinical stage Ta at screening, *n* (%)	17 (100)
Concomitant CIS at screening, *n* (%)	0 (0)
WHO Low-Grade 2004/2016 at screening, *n* (%)	12 (71)
Prior intravesical therapies, *n* (%)	
Mitomycin	4 (24)
Gemcitabine	3 (18)
Thiotepa	1 (5.9)
BCG	3 (18)
BCG (TICE)	1 (5.9)

BCG = Bacillus Calmette-Guérin; NMIBC = Non-Muscle-Invasive Bladder Cancer; WHO = World Health Organization. Estimates were given as medians or frequencies.

**Table 2:** Summary of lesion-level outcomes in efficacy-evaluable participants by IBCG risk group (*n* = 10)

**IBCG risk group**	**Cohort (dose)**	**Participant ID**	**Tumor status**	**Tumor grade at treatment**	**Prior failed therapies**	**Response in the treated tumor** **(CR/tumor shrinkage/none)**	**Response in nontreated tumors (CR/tumor shrinkage/none)**
**IBCG Intermediate Risk**	4a (100 µg)	106-002^a^	Multiple	Low-grade	Yes (Mitomycin C, Gemcitabine)	CR	CR (2/2 Tumors)
	4a (100 µg)	109-001^a^	Multiple	Low-grade	Yes (Incomplete BCG)	CR	CR (1/2 Tumors)
	4a (100 µg)	102-004 ^a^	Multiple	Low-grade	No	CR	CR (1/1 Tumors)
	4b (100 µg)	115-002	Multiple	Low-grade	No	Tumor shrinkage	None (0/1 Tumors)
	4c (200 µg)	113-002^a^	Multiple	Low-grade	Yes (Gemcitabine)	CR	None (0/1 Tumors)
**IBCG** **High Risk**	4a (100 µg)	108-001	Single	High-grade	Yes (Mitomycin C)	Tumor shrinkage	Single Tumor Only
	4b (100 µg)	105-003	Multiple	High-grade	Yes (BCG, Gemcitabine)	Tumor shrinkage	None (0/1 Tumors)
	4b (100 µg)	108-002	Single	High-grade	No	None	Single Tumor Only
	4c (200 µg)	115-003^a^	Multiple	High-grade	N0	Tumor shrinkage	Single Tumor Only
	4c (200 µg)	103-001	Multiple	Low-grade	Yes (BCG)	CR (1/2 Tumors in injected field)	CR (1/3 Tumors)

Footnote: Local pathology with no evidence of carcinoma in 3/3 target specimens. Central pathology demonstrated a single fibrovascular core in 1/3 target specimens, consistent with a small area of papillary disease of unclear distance from the target injection. Patients selected for immune fluorescence staining and analysis are highlighted with “^a^”. BCG = Bacillus Calmette-Guérin; IBCG = International Bladder Cancer Group; CR = Complete Response.

Seventeen participants received a single focal bel-sar injection (100–200 µg) followed by TURBT 7–12 d later: five participants received bel-sar without light activation as an initial safety cohort; 12 subsequently received light activation approximately 24 h postinjection. Safety oversight was provided by a safety review board.

Bel-sar was administered in an office-based setting via flexible white-light cystoscopy using a 23-gauge injection needle to inject a single target lesion. When multiple tumors were present, the largest lesion was injected with 100 µg (cohorts 4a, 4b) or 200 µg (cohort 4c). Cystoscopic light activation was performed using 689-nm laser illumination of the injected lesion 24 h later, with standardized fluence and exposure time based on lesion surface area. Participants were monitored for safety for 56 (+7) d. Tumor response was assessed by central pathology, with complete response (CR) defined as absence of urothelial carcinoma in the resected lesion.

### 2.2 Samples and participants selection for multiplex immunofluorescence

Exploratory and hypothesis-generating immune biomarker analyses were performed in accordance with Reporting Recommendations for Tumor Marker Prognostic Studies (REMARK) guidelines [10]. Analyses were restricted to tumors demonstrating clinical activity after light-activated bel-sar, precluding comparisons with nonresponding lesions or formal assessment of resistance mechanisms. Five participants (four IR NMIBC, one HR NMIBC) with available paired pre- and post-treatment tissue were selected based on evidence of tumor response (CR or tumor size reduction). Overall, 27 formalin-fixed paraffin-embedded (FFPE) specimens from treated and untreated lesions were analyzed by multiplex immunofluorescence (Supplementary Table 1).

### 2.3 Multiplex immunofluorescence on tumor specimens

Multiplex immunofluorescence was performed using Paletrra technology (NeoGenomics Laboratories, Fort Myers, FL, USA) with a 24-marker immune panel spanning more than 30 coexpressing cell types (Supplementary Tables 2–3), including lymphoid, myeloid, activation, and checkpoint markers. FFPE sections underwent iterative staining with paired cyanine dye–labeled antibodies, sequential imaging, and dye inactivation as previously described [11]. Cell densities were quantified and normalized to tissue area (cells/mm^2^).

Image segmentation and immune cell phenotyping were conducted using an artificial intelligence (AI)-assisted platform based on marker coexpression (Paletrra AI). Spatial neighborhood clustering was performed using K-means–based algorithms to visualize treatment-associated immune architecture. All analyses underwent standardized quality control procedures.

### 2.4 Quantification of eosinophils in hematoxylin and eosin-stained NMIBC FFPE tissue

Whole-slide hematoxylin and eosin (H&E) images of NMIBC FFPE specimens were scanned using a Zeiss slide scanner (40× magnification; StageBio LLC, Frederick, MD, USA) and analyzed with an AI-assisted image analysis platform (Indica Halo platform, v3.6; NeoGenomics Laboratories). Tissue artifacts were excluded during tissue segmentation. Eosinophils were quantified using a trained classifier with pathologist review for accuracy.

### 2.5 Statistical analysis

This study was designed for safety, feasibility, and biological characterization; it was not powered for formal hypothesis testing. Analyses are descriptive and exploratory. The safety population included all participants receiving bel-sar; efficacy-evaluable participants received protocol-defined treatment and had available post-treatment histologic assessment. For preliminary efficacy, CR of the treated lesions was displayed descriptively and summarized as a relative proportion.

Exploratory immune biomarker analyses were performed to evaluate associations between immune cell densities and tumor response in treated lesions and were restricted to participants with adequate pre- and/or post-treatment tissue demonstrating clinical activity (CR or tumor size reduction). Tumor response was coded as a binary variable (CR = 1; pretreatment or nonresponse = 0). Cell densities were summarized descriptively; associations between immune cell densities and CR were explored using nonparametric Spearman rank correlations because of the small sample size and nonnormal distributions. Data from one participant (113-002) were excluded because of insufficient cell counts. No imputation was performed for missing tissue. Statistical analyses were performed using GraphPad Prism (version 10.4.1).

## 3 Results

### 3.1 Baseline characteristics

Seventeen participants with visible papillary stage Ta NMIBC were enrolled as of August 1, 2025 (Table 1). Median age was 72 yr, and 82% were male. Most participants had recurrent disease (71%) and multiple tumors (53%), with tumor size <3 cm in 77%. TURBT was performed 7–12 d after treatment per protocol. Of 17 treated participants, 12 received light activation. Ten were included in the efficacy-evaluable population; two were excluded due to absence of tumor on on-treatment biopsy (despite visible disease and prior biopsy-confirmed low-grade NMIBC) or incomplete dose delivery.

Patients were classified according to the AUA NMIBC risk stratification (2020), with efficacy analyses conducted using the IBCG system [8], [9]. One participant was prospectively reclassified from IBCG intermediate- to high-risk based on multiple adverse features, including BCG failure. Twelve patients (71%) had low-grade tumors by WHO 2004/2016 criteria, and none had concomitant carcinoma in situ. Prior intravesical therapy included mitomycin (*n* = 4), gemcitabine (*n* = 3), and BCG (*n* = 4).

### 3.2 Safety

As of August 1, 2025, all 17 enrolled participants were evaluable for safety. Eleven (65%) experienced at least one treatment-emergent adverse event (TEAE), including five (30%) with events related to bel-sar, injection, or laser procedures. All related TEAEs were grade 1 and limited to urinary symptoms (dysuria, urgency, nocturia, or hematuria). No grade ≥2 TEAEs, serious adverse events, or dose-limiting toxicities were observed (Table 2).

### 3.3 Preliminary efficacy

Ten participants treated with light-activated bel-sar (five IR and five HR per IBCG criteria) were evaluable for efficacy (Table 2).

**Fig. 2:**
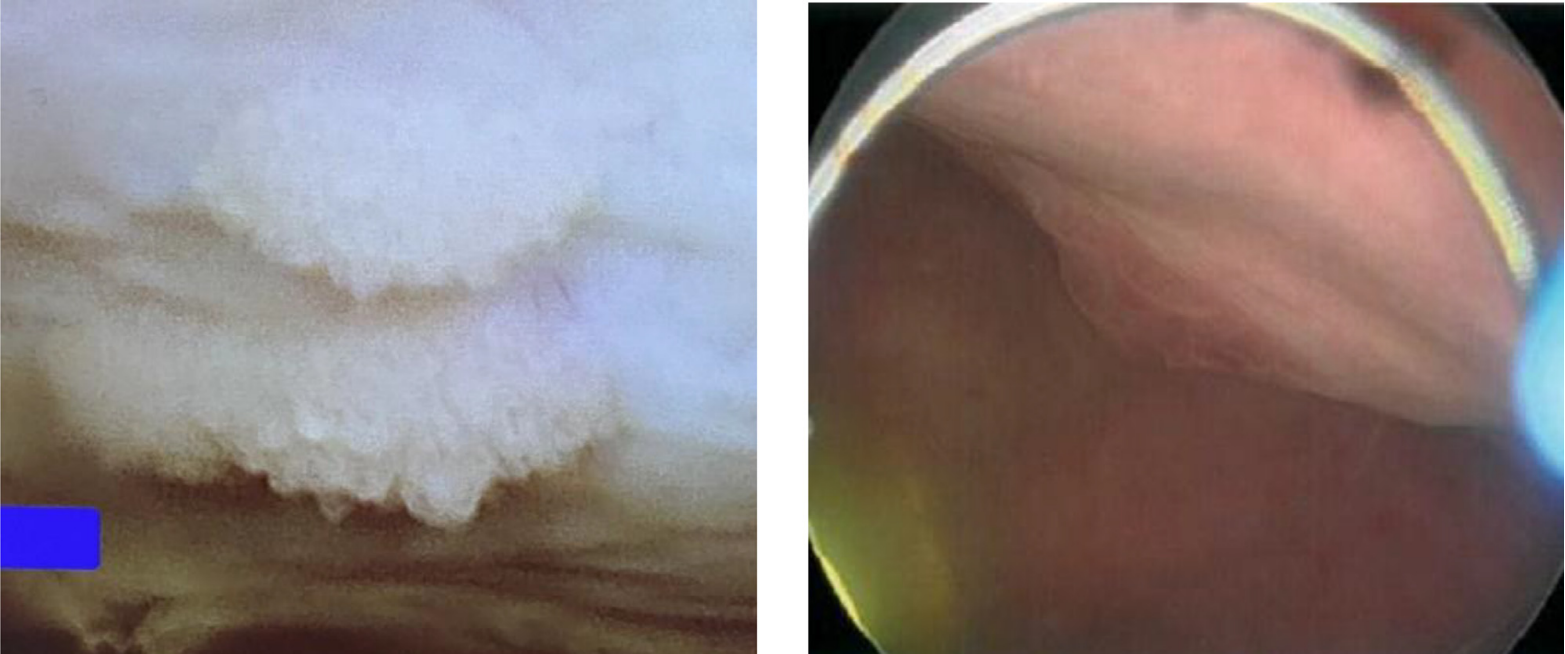
Representative cystoscopic views of a bladder tumor at baseline (left) and at TURBT 8 d after AU-011 treatment (right), demonstrating complete ablation (IBCG intermediate-risk NMIBC). IBCG = International Bladder Cancer Group; TUBRT = Transurethral Resection of Bladder Tumor

Among IR participants, 4/5 (80%) achieved CR in the treated lesion, defined by the absence of viable tumor on TURBT pathology; the remaining participant demonstrated tumor size reduction on cystoscopy (Fig. 2). Notably, CRs were also observed in nontreated lesions in three of five participants, suggesting a potential urothelial field effect.

Among HR participants, 3/5 (60%) demonstrated visual regression of the treated lesion, and 1/5 (20%) achieved a histologically confirmed CR in both the treated lesion and one of three nontreated lesions. Overall, 4/5 (80%) showed either tumor size reduction or CR.

### 3.4 Immune response assessment

Matched pre- and post-treatment specimens were analyzed to characterize treatment-associated changes in the tumor immune microenvironment. Immune profiling was restricted to tumors demonstrating clinical activity, precluding comparisons with nonresponding lesions or formal assessment of resistance mechanisms; therefore, analyses should be considered hypothesis-generating. Where paired baseline tissue was unavailable, post-treatment specimens were analyzed descriptively and not interpreted as longitudinal changes. Multiplex immunofluorescence using a 24-marker panel was performed on 27 slides from five participants with CR or visual tumor size reduction [12], enabling normalized comparison of immune cell densities.

#### 3.4.1 Global immune profiles

**Fig. 3:**
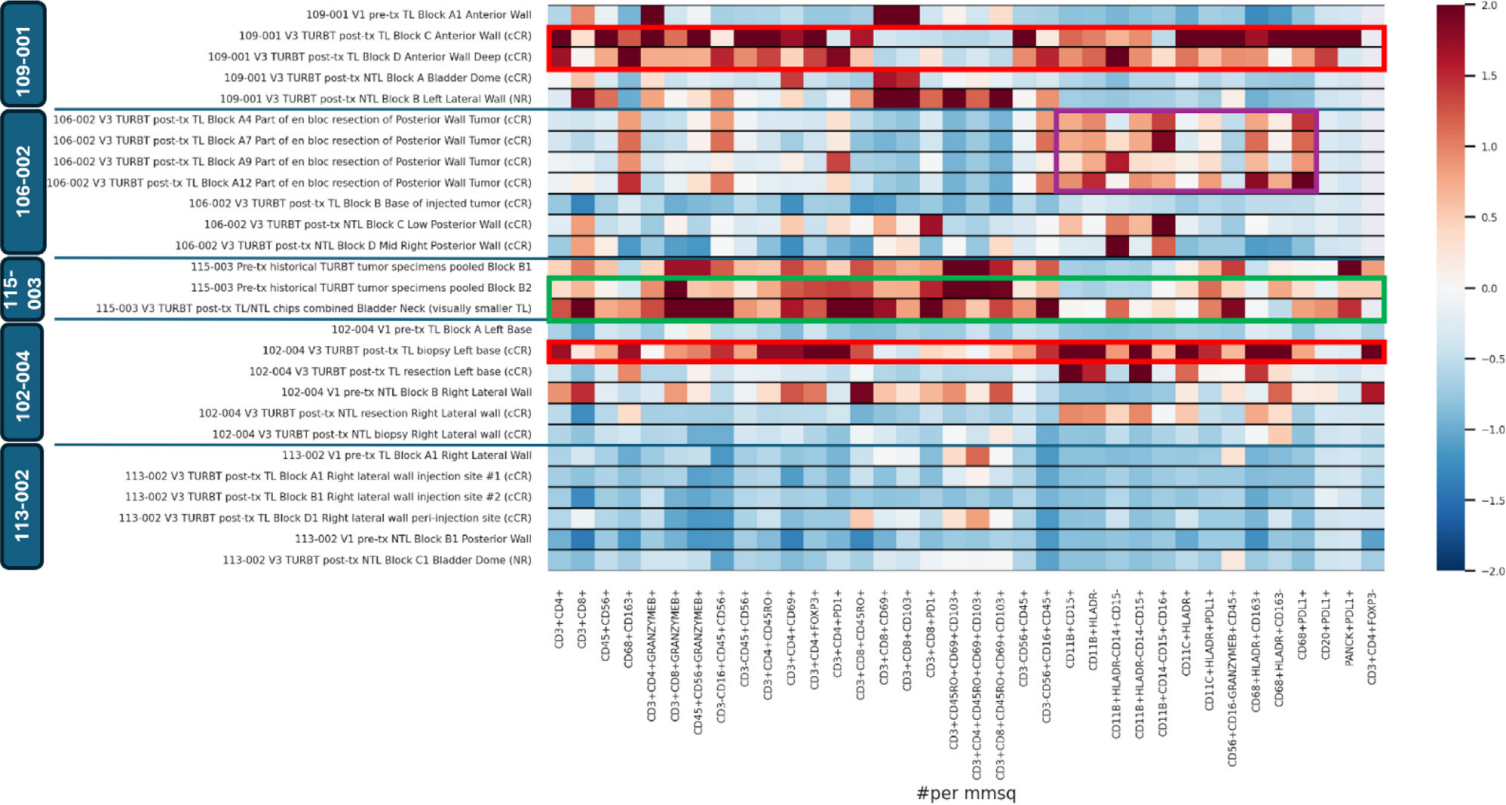
Heatmap of global immune profiles in the tumor tissue of five participants with CR or visual tumor shrinkage, depicting z-scale normalized expression of cell density (defined as cell count/mm2 of tissue on the slide) of key coexpressing cell types (columns). cCR = Clinical Complete Response; CR = Complete Response; NR = No Response; NTL = Nontreated Lesion; TL = Treated Lesion; TURBT = Transurethral Resection of Bladder Tumor. The color scale bar shows the gradient of cell density, from low (blue) to high (red) cell. A total of 27 samples across the 5 selected participants are listed in individual rows in sequential order, first by participant, then by treated versus non-treated lesions, and by pre-treatment and post-treatment status (where samples were available). Samples, in individual rows, were named according to this format: Subject ID, visit number, and treatment status (either V1 pre-treatment, or V3 TURBT post-treatment), TL or NTL, Specimen Block ID (eg. A1), anatomical location of the lesion, and response (cCR) or NR. Bel-sar converted immune cold tumors into hot lesions in two patients (109-001, 102-004, red rectangles) and reversed immune exhaustion in one patient (115-003, green rectangle). Participant 106-002 showed a predominantly myeloid phenotype (purple rectangle), without baseline tissue for reference. Participant 113-002, whose TURBT was day 17 post-bel-sar treatment, displayed evidence of immune resolution.

Heatmap analysis (Fig. 3) showed conversion of immune-cold to hot lesions in two participants and reversal of immune exhaustion in one, with increased lymphoid and myeloid infiltration. One participant displayed a predominantly myeloid phenotype after treatment, while another, sampled later, showed immune resolution with de novo formation of mature tertiary lymphoid structures (TLS) despite low effector cell densities.

#### 3.4.2 Neighborhood clustering

**Fig. 4:**
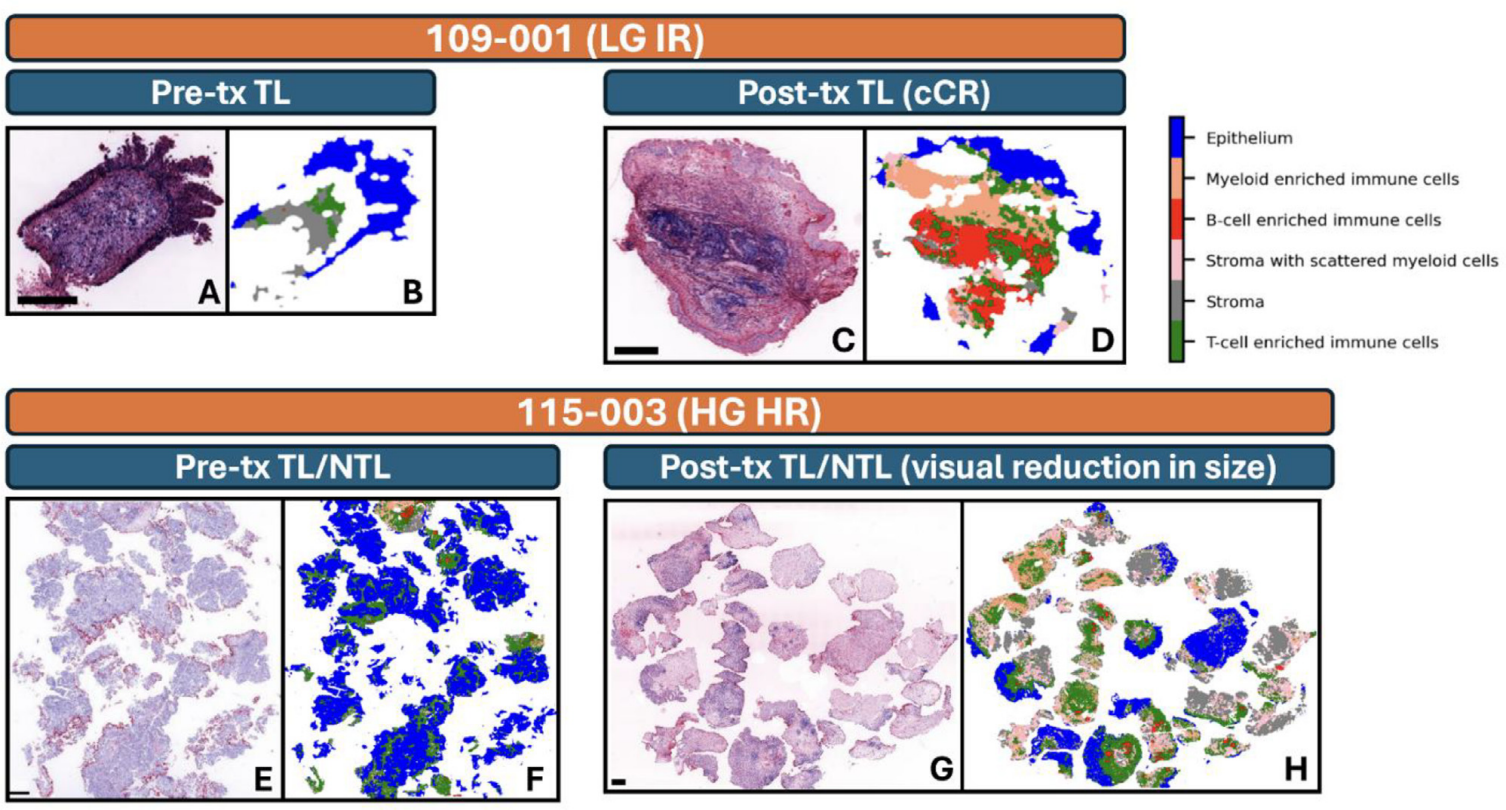
Neighborhood clustering analysis. LG = Low Grade; HG = High Grade; IR = Intermediate Risk; NTL = Nontreated Lesion; TL = Treated Lesion; TURBT = Transurethral Resection of Bladder Tumor; Tx = Treatment; post-tx = Post-treatment; pre-tx = Pretreatment; cCR = Complete Clinical Response; NR = No Response. Images of two representative participants, one with LG IR disease (109-001, top row, treated lesion) and one with HG HR disease (115-003, bottom row, treated and nontreated lesions combined in the same specimen). Panels A, C, E, and G are virtual H&E images, with adjacent panels B, D, F, and H, corresponding to overlays showing neighborhood cluster distribution across the sample. For participant 109-001, TURBT occurred 9 days post-bel-sar treatment (Panel C and D). The black scale bars shown for Panels A-D are 500 μm. For participant 115-003, TURBT occurred 16 d post-bel-sar treatment (Panel G and H). The black scale bars shown for Panels E–H are 1 mm. The colored legend on the top right depicts the 6 annotated immune cell-enriched areas that the unsupervised clustering algorithm identified based on biomarker prevalence.

**Fig. 5:**
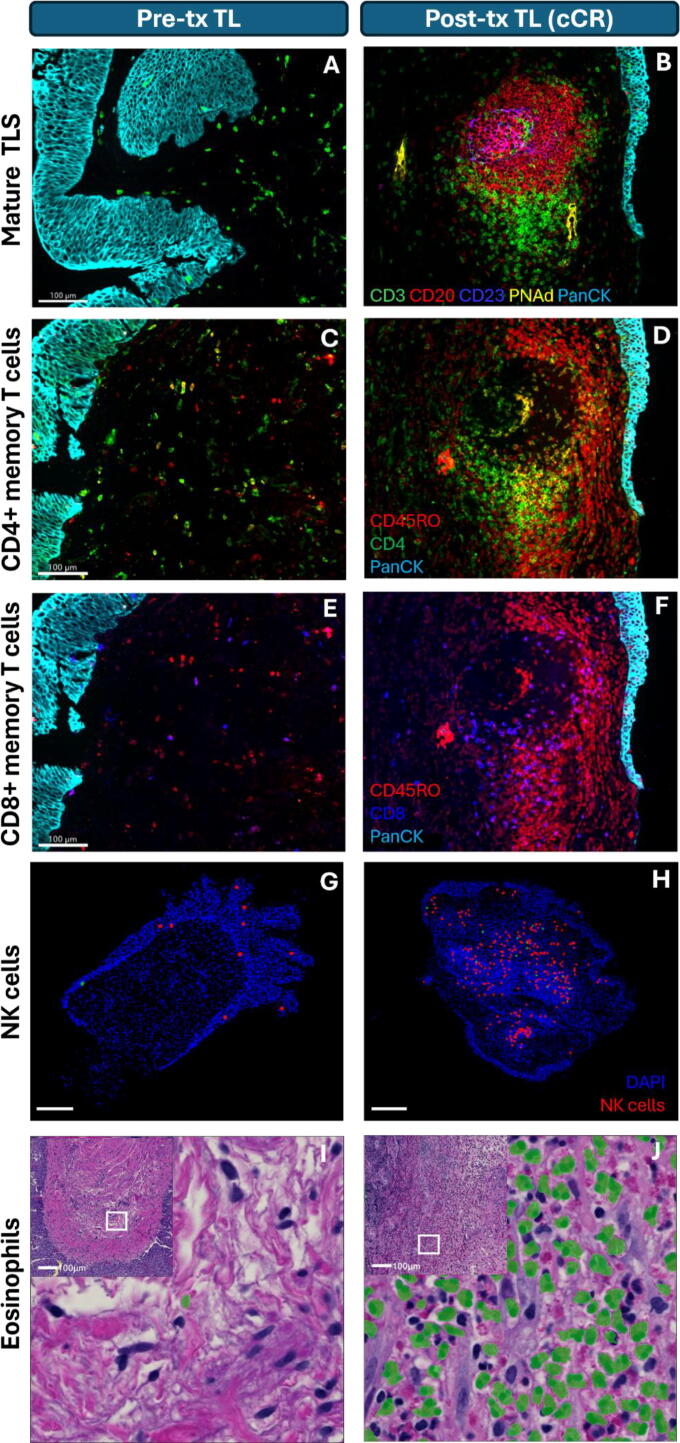
Representative images from pre- and post-treatment lesions (TL) in which a clinical complete response (cCR) was observed. cCR = Clinical Complete Response; LG = Low Grade; IR = Intermediate Risk; NK cell = Natural Killer Cells; TL = Treated Lesion; TLS = Tertiary Lymphoid Structures; tx = Treatment. Multiplex immunofluorescence images (A–F) from participant 102-004 and analytical images (G–J) from participant 109-001, both with LG IR disease, display mature TLS (A-B), CD4+ memory T cells (C–D), and CD8+ memory T cells (E–F). Mature TLS were identified by a well-defined CD20hi germinal center with extensive or isolated CD23+ follicular dendritic cell staining (A–B). CD4+ memory T cells (CD4+ CD45RO+ costained) were identified as yellow cells (C–D). CD8+ memory T cells (CD8+ CD45RO+ costained) were identified as magenta cells (E–F). NK cells (CD45+ CD56+ co-stained) were identified analytically and depicted as red cells (G–H). Eosinophils were identified using the HALO-classifier and depicted as green cells (I–J). The white scale bars are 100 μm in A-F, 250 μm in G, 500 μm in H, and 100 μm in the inset images in I-J.

Neighborhood clustering analysis was used to visualize immune cell-enriched regions within tumor sections (Fig. 4). Six distinct clusters were identified and quantified by relative slide area. Following bel-sar treatment, myeloid-, B-, and T-cell-enriched clusters—largely absent at baseline—were consistently induced. In representative cases, immune-enriched regions expanded substantially after treatment, replacing tumor-dominant areas (eg, from 6% to >80% in one participant and from 23% to 52% in another) (Fig. 4), consistent with conversion toward an inflamed, immune-active phenotype (see Fig. 5).

#### 3.4.3 Tertiary lymphoid structures

Tumor-associated tertiary lymphoid structures (TLS) were identified and quantified using canonical markers for B cells (CD20), T cells (CD3), follicular dendritic cells (CD23), and high endothelial venules (PNAd) (Supplementary Table 4). Mature TLS were defined by the presence of CD23+ follicular dendritic cell networks. Although mature TLS are infrequently observed in NMIBC [13], they were detected in post-treatment specimens from all five participants (Supplementary Table 4). In three of four participants with paired biopsies, mature TLS were absent at baseline and formed de novo following bel-sar treatment. Despite variability in size and architecture, their emergence is consistent with the induction of localized adaptive immune microenvironments.

#### 3.4.4 T-cell infiltration

Tumor sections were analyzed for CD4+ conventional T cells (CD3+ CD4+ Foxp3- “Tconv”), regulatory T cells (CD3+ CD4+ Foxp3+ “Treg”), CD8+ T cells, and immune memory subsets. Robust recruitment of CD4+ Tconv cells was observed in both treated and untreated lesions, accompanied by increased expression of activation markers (CD69, PD-; data not shown). Consistent with TLS formation, CD4+ Tconv densities increased markedly in treated lesions (7.3-fold, 4.9-fold, and 1.8-fold in three participants; Fig. 4D). CD8+ T-cell infiltration was less pronounced (Supplementary Table 4), consistent with emerging evidence identifying CD4+ T cells as dominant cytotoxic effectors in bladder cancer [14], [15]. Although Treg levels increased after treatment (Fig. 4F), CD4+ Tconv cells consistently outnumbered Tregs, resulting in favorable Tconv:Treg ratios (3–5 across lesions).

#### 3.4.5 Immune memory

CD45RO was used to identify memory T cells within tumor tissue [16]. Both CD4+ and CD8+ memory T cells were detected in all five participants, including within TLS, indicating induction of tumor-associated immune memory following bel-sar treatment (Supplementary Table 4).

#### 3.4.6 Innate immune effectors

Innate immune populations contributed prominently to the bel-sar-associated immune response. At 9–17 d post-treatment, antigen-presenting cells, granulocytes, and innate cytotoxic effectors remained abundant, consistent with a sustained innate activation. Dendritic cells, neutrophils, eosinophils, and natural killer (NK) cells were enriched in both treated and untreated lesions, with higher densities in treated tumors. Notably, NK cells and eosinophils demonstrated marked post-treatment expansion, supporting prolonged innate immune activation accompanying adaptive immune responses (Supplementary Table 4)

#### 3.4.7 Regulatory and reparative populations

Reparative macrophages (CD68⁺HLA-DR⁺CD163⁺) and granulocytic myeloid-derived suppressor cells (G-MDSCs; CD11b⁺HLA-DR⁺CD14⁻CD15⁺) were observed post-treatment, consistent with immune resolution and urothelial repair following tumor clearance (Supplementary Table 4).

#### 3.4.8 Correlation of immune response with clinical response

**Fig. 6:**
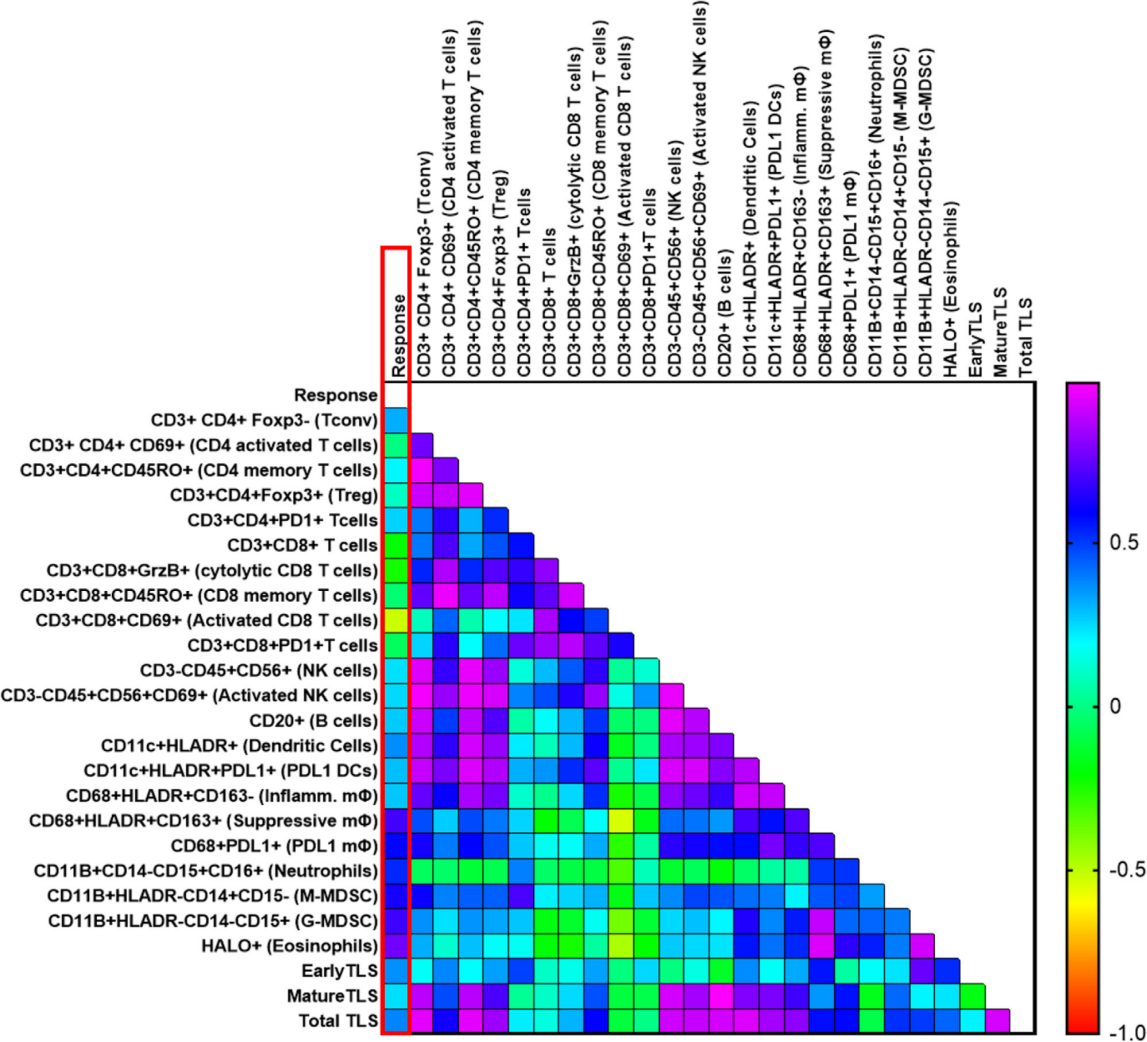
Correlation matrix of selected cell types and response. cCR = Clinical Complete Response; pre-tx = Pretreatment; NR = No Response; GraphPad Prism = Software for statistical analysis and graphing. The Spearman Rank correlation matrix was generated to assess relationships between key coexpressing cell types and tumor response in the treated lesions. Response was binary: cCR = 1, pre-tx/NR = 0. Cell density parameters were compared in Graphpad Prism using a column table format. Data from participant 113-003 were excluded due to low immune cell counts to avoid confounding the analysis. To verify R values, logistic regression analyses (not shown) were performed with cell type as the predictor and binary response outcome. The color scale spans -1.0 (red, negative association) to +0.99 (magenta, positive association). The matrix is symmetrical, with diagonal cubes = 1.0; diagonal and upper-right cells were whited out for clarity. The first column (red rectangle) shows the correlation of each cell type with clinical response.

Exploratory correlation analyses were performed to assess associations between immune cell populations and tumor response following bel-sar treatment. CR was associated with eosinophils, myeloid populations (macrophages, dendritic cells, and MDSCs), TLS presence, and cytotoxic effector cells, including NK cells and CD4+ Tconv T cells (Fig. 6, first column).

Collectively, multiplex immunofluorescence demonstrated coordinated tumor ablation and immune activation characterized by TLS formation, expansion of cytotoxic and memory CD4⁺ T cells, and recruitment of NK cells and eosinophils. These findings support bel-sar’s dual mechanism integrating direct cytotoxicity with subsequent innate and adaptive immune activation.

## 4 Discussion

In this first-in-human evaluation, focal bel-sar treatment was feasible, well tolerated, and showed encouraging preliminary efficacy in IR and HR NMIBC. CRs were observed not only in injected but also in untreated lesions, suggesting a pan-urothelial field effect. Tumor regression within 7–12 d after a single light-activated dose supports rapid cytotoxicity coupled with immune activation, consistent with bel-sar’s proposed dual mechanism of action.

Current NMIBC management relies on TURBT followed by intravesical BCG or chemotherapy. Immune activation following BCG has been well documented, including the presence of activated lymphocytes in urine [17]. More recent work has further characterized BCG-induced immune responses, highlighting both their complexity and temporal dynamics [14], [18], [19], [20]. In contrast to intravesical therapies and novel delivery systems that rely on repeated dosing to maintain efficacy [21], [22], bel-sar induces rapid local tumor ablation and immunogenic cell death, releasing tumor-specific neoantigens and damage-associated molecular patterns that trigger coordinated innate and adaptive immune responses.

Exploratory immune profiling demonstrated the ability of bel-sar to coordinate activation of innate and adaptive effectors, including myeloid, B-cell, and T-cell infiltration, together with the conversion of immune-cold into immune-active lesions and early formation of mature TLS. Recent evidence identified TLS and activated B-cell niches as key organizers of antitumor immunity in urothelial cancer, reflecting an inflamed, antigen-rich tumor microenvironment associated with response to immunotherapy [23], [24], [25], [26]. Their emergence within days further supports bel-sar’s potential for durable immune surveillance and protection from recurrence.

**Fig. 7:**
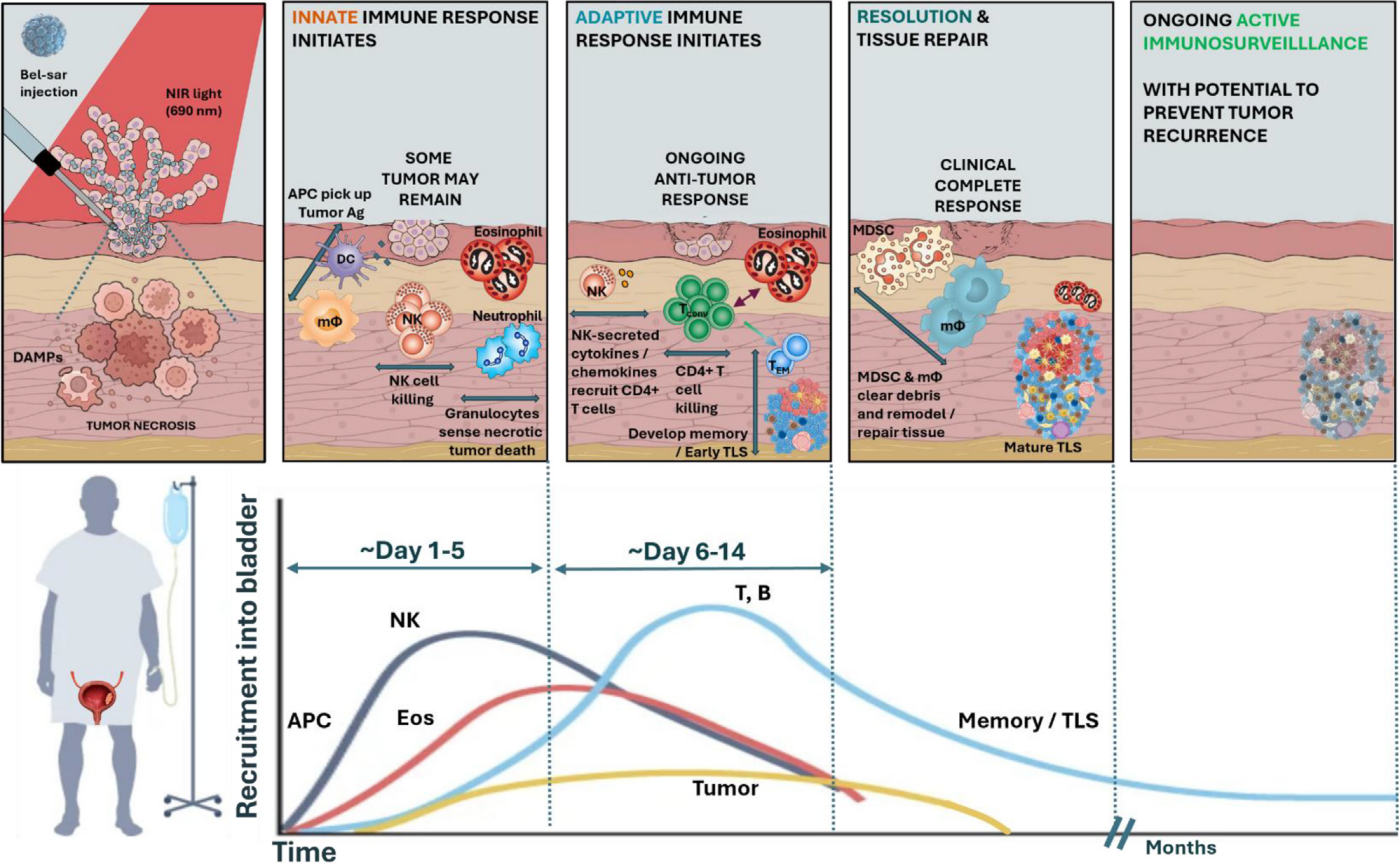
Proposed, hypothesis-generating model of bel-sar mechanism of action in bladder cancer. APCs = Antigen-Presenting Cells; CD4+ Tconv cells = CD4-positive Conventional T cells (here: cytolytic subset in bladder cancer); CD8+ T cells = Cytotoxic T lymphocytes (killer T cells); DAMPs = Damage-Associated Molecular Patterns; DCs = Dendritic Cells; G-MDSCs = Granulocytic Myeloid-Derived Suppressor Cells; IFN-γ = Interferon gamma; IL = Interleukin; IP-10 = Interferon gamma-induced protein 10 (also called CXCL10); MIG = Monokine Induced by Gamma interferon (also called CXCL9); MIP-1α = Macrophage Inflammatory Protein 1 alpha (also called CCL3); MOA = Mechanism of Action; NK cells = Natural Killer cells; RANTES = Regulated upon Activation, Normal T cell Expressed and Secreted (also called CCL5); ROS: Reactive Oxygen Species; TLS = Tertiary Lymphoid Structures. Based on early clinical and exploratory immune profiling data from this Phase 1 study and prior preclinical research, bel-sar is hypothesized to exert antitumor activity through a combination of direct tumor ablation and subsequent immune activation. Upon light activation, bel-sar triggers a ROS cascade, causing tumor necrosis and release of DAMPs. These events are proposed to activate an immune response, inducing APCs, NK cells, neutrophils, and eosinophils, as observed in post-treatment specimens. Granulocytes may amplify anti-tumor immunity through perpetuation of the ROS cascade. NK cells kill tumor cells as well as secrete cytokines and chemokines, driving T-cell infiltration. CD8+ T cells appear to contribute to the anti-tumor immune response; however, CD4+ Tconv cells expand, infiltrate, and likely mediate the majority of tumor killing. Memory T cells and TLS emerge in situ. As inflammation resolves, macrophage populations appear to shift toward tissue repair, with the presence of G-MDSCs. Eosinophils and CD4+ Tconv likely engage in active crosstalk; the eosinophils sustained by cytokines produced by CD4+ Tconv cells, in turn, promote memory and mature TLS formation for long-term immunosurveillance. Functional relevance, durability of these interactions, and predictive value for treatment response of these early changes remain to be established. This schematic represents a conceptual framework and should be interpreted as hypothesis-generating rather than definitive.

The predominance of CD4+ over CD8+ T cells aligns with emerging evidence of their functional relevance in bladder cancer [14], [15]. Marked eosinophil recruitment—rare in NMIBC—was associated with CR, supporting a potential role as an immune amplifier in bel-sar–mediated antitumor activity. Emerging evidence indicates that eosinophils can support immunity through cytokine release, modulation of myeloid and lymphoid populations, facilitation of immune cell trafficking, and contribution to TLS formation. Collectively, these findings support a model of direct tumor ablation accompanied by coordinated innate and adaptive immune activation (Fig. 7).

Study limitations include the small sample size and short follow-up, which preclude conclusions regarding long-term immune surveillance, immune durability, or sustained clinical benefit, despite the observation of immune memory markers and TLS. Exploratory immune analyses were restricted to lesions showing clinical activity and are therefore subject to selection bias and cannot be used to distinguish responders from nonresponders. Nonresponding lesions were not analyzed, limiting the ability to assess resistance mechanisms or generalize immune patterns across the full treated population. In addition, expanded immune profiling in future studies will be required to further define interactions between eosinophils and innate-like lymphocytes such as γδ T cells.

## 5 Conclusions

Bel-sar is a first-in-class VDC combining selective tumor ablation with immune activation in NMIBC. The feasibility of focal administration, early efficacy signals, and induction of coordinated immune responses support further evaluation as a front-line immune-ablative or neoadjuvant strategy with reduced treatment burden.

The ongoing phase 1b/2 study will assess repeat dosing, higher doses, and longitudinal immune correlatives to determine whether these features translate into durable clinical benefit relative to existing standards.

  ***Author contributions:*** Sabine D. Brookman-May had full access to all the data in the study and takes responsibility for the integrity of the data and the accuracy of the data analysis.

  *Study concept and design:* Brookman-May, Hopkins, McQuaid, Penny, De los Pinos,

Lerner, Schiller, Kines.

*Acquisition of data:* Lerner, Shore, Linehan, Agarwal.

*Analysis and interpretation of data:* Brookman-May, Penny, McQuaid.

*Drafting of the manuscript:* Brookman-May, Penny, McQuaid.

*Critical revision of the manuscript for important intellectual content:* Brookman-May, Penny, McQauid, De los Pinos, Hopkins, Shore, Kates, Jacob, Kines, Schiller, Linehan, Agarwal, Lerner.

*Statistical analysis:* Penny, Brookman-May, McQuaid.

*Obtaining funding:* Brookman-May, McQuaid.

*Administrative, technical, or material support:* Penny, Brookman-May, McQuaid.

*Supervision:* Brookman-May.

*Other:* None.

  ***Financial disclosures:*** Sabine Brookman-May, Xianne Penny, Joseph McQuaid, Jill Hopkins, Eli de los Pinos are employees of Aura Biosciences Inc., Boston, MA, USA. Rhonda Kines and John Schiller were mainly involved in the development of AU-011/bel-sar through their work and during employment at the NIH. Neal Shore, Jenifer Linehan, Seth Lerner, and Piyush Agarwal are investigators of the reported study. Max Kates and Joseph Jacob are advisors for Aura Biosciences Inc.

  ***Funding/Support and role of the sponsor:*** Aura Biosciences Inc., Boston, MA, USA is the sponsor of this study.

  ***Acknowledgements:*** We gratefully acknowledge Dr Scott Lucia for serving as the central pathologist for this study. We also thank Tish Webb, Lindsay Laudal, and Heather Mann of Aura Biosciences for their support of study operations and site activities for the AU-011-102 trial. In addition, we acknowledge the team at NeoGenomics Inc., including Sandra Lam, Harry Nunns, Jeffrey Lock, Qingyan Au, Jiong Fei, and Erinn Parnell, for their contributions to multiplex immunofluorescence staining and digital image analysis, including eosinophil classifier development.

## Appendix A Supplementary material

Supplementary Data 1 Immunoflueorescence information including corresponding markers and co-expressing cell types.
