# Supplementary material for: Early Efficacy and Exploratory Biomarker Data of Bel-sar in Patients with Intermediate-risk and High-risk Non–muscle-invasive Bladder Cancer
Source: Eur Urol Open Sci. 2026 Jun 13;89:77–88. doi: 10.1016/j.euros.2026.05.017 (PMC13279200; doi:10.1016/j.euros.2026.05.017)
Supplement: Supplementary Data 1 — Immunoflueorescence information including corresponding markers and co-expressing cell types. [file mmc1.docx]

**Supplementary Table 1 - Samples analyzed by multiplex immunofluorescence**

|  |  | **TL (treated lesion)** | | **NTL (non-treated lesion)** | |  |
| --- | --- | --- | --- | --- | --- | --- |
| **Participant ID** | **NMIBC Diagnosis** | **Pre-tx** | **Post-tx** | **Pre-tx** | **Post-tx** | **TURBT**  **(days post V1)** |
| 109-001 (A3) | Low-Grade Intermediate Risk | ✓ | ✓ | Not available | ✓ | 9 d |
| 106-002 (A1) | Low-Grade  Intermediate Risk | Not available | ✓ | Not available | ✓ | 9 d |
| 115-003 (C2) | High-Grade  High Risk | ✓  (TL/NTL pooled) | ✓  (TL/NTL pooled) | - | - | 16 d |
| 102-004 (A4) | Low-Grade  Intermediate Risk | ✓ | ✓ | ✓ | ✓ | 9 d |
| 113-002 (C1) | Low-Grade  Intermediate Risk | ✓ | ✓ | ✓ | ✓ | 17 d |
| NMIBC: Non-Muscle Invasive Bladder Cancer; NTL: Non-Treated Lesion; TL: Treated Lesion; tx: treatment TURBT: Transurethral Resection of Bladder Tumor. Note: Sampling and immune profiling was limited to participants treated with light-activated bel-sar who demonstrated clinical activity (complete response or tumor size reduction) and for whom adequate pre- and/or post-treatment tissue was available, precluding comparative analyses with non-responding lesions or formal investigation of resistance mechanisms. | | | | | | |

**Supplementary Table 2 - 24-plex Custom Marker Panel**

| **No.** | **Marker** | **To identify** |
| --- | --- | --- |
| 1 | CD20 | B cells; TLS |
| 2 | CD56 | NK cells |
| 3 | CD15 | Neutrophils, MDSC |
| 4 | CD103 | Resident Memory T cells |
| 5 | PanCK | Tumor cells |
| 6 | PNAd | High Endothelial Venule |
| 7 | CD4 | CD4+ T cells |
| 8 | HLA-DR | MHC class II |
| 9 | CD8 | CD8+ T cells |
| 10 | CD16 | NK cells; neutrophils |
| 11 | CD3 | T cells |
| 12 | CD23 | Follicular Dendritic Cells |
| 13 | CD68 | Macrophages |
| 14 | CD45RO | Memory |
| 15 | Granzyme B | Cytolytic effector cells |
| 16 | CD163 | Macrophage; monocytes |
| 17 | CD45 | Hematopoietic cells |
| 18 | PD-L1 | Expression on myeloid and tumor cells |
| 19 | CD14 | MDSC; monocytes |
| 20 | Foxp3 | Tregs |
| 21 | CD11c | Dendritic Cells |
| 22 | CD69 | Activated T cells; Resident memory T cells |
| 23 | PD1 | Activation |
| 24 | CD11b | MDSC; neutrophils |
| CD: Cluster of Differentiation; HLA: Foxp3: Forkhead box protein P3; Human Leukocyte Antigen; HLA-DR: Human Leukocyte Antigen – DR isotype; PanCK: Pan Cytokeratin; PNAd: Peripheral Node Addressin; PD1: Programmed Death-1; PD-L1: Programmed Death-Ligand 1 | | |

**Supplementary Table 3: Co-expressing cell types and the corresponding markers**

| **No.** | **Co-expression** | **Phenotype** |
| --- | --- | --- |
| ***CD4+ T cells*** | | |
| 1 | CD3+ CD4+ | Total CD4+ T cells |
| 2 | CD3+ CD4+ Foxp3- | Total CD4+Tconv cells |
| 3 | CD3+ CD4+ GrzB+ | CD4+ Tconv cytolytic |
| 4 | CD3+ CD4+ CD45RO+ | CD4+ memory T cells |
| 5 | CD3+ CD4+ CD69+ | Activated CD4+ Tconv cells |
| 6 | CD3+ CD4+ Foxp3+ | Treg |
| 7 | CD3+ CD4+ PD1+ | PD1-expressing CD4+ T cells |
| ***CD8+ T cells*** | | |
| 8 | CD3+ CD8+ | Total CD8+T cells |
| 9 | CD3+ CD8+ GrzB+ | CD8+ T cells cytolytic |
| 10 | CD3+ CD8+ CD45RO+ | CD8+ memory T cells |
| 11 | CD3+ CD8+ CD69+ | Activated CD8+ T cells |
| 12 | CD3+ CD8+ PD1+ | PD1-expressing CD8+ T cells |
| ***Resident Memory T cells*** | | |
| 13 | CD3+ CD4+ CD45RO+ CD69+ CD103+ | Resident memory CD4+ T cells |
| 14 | CD3+ CD4+ CD45RO+ CD103+ | Resident memory CD4+ T cells w/o CD69 |
| 15 | CD3+ CD8+ CD45RO+ CD69+ CD103+ | Resident memory CD8+ T cells |
| 16 | CD3+ CD8+ CD45RO+ CD103+ | Resident memory CD8+ T cells w/o CD69 |
| 17 | CD3+ CD45RO+CD69+CD103+ | Total Resident memory T cells |
| ***NK cells*** | | |
| 18 | CD3- CD45+ CD56+ | NK cells |
| 19 | CD3- CD45+ CD56+ CD69+ | Activated NK cells |
| 20 | CD3- CD16+ CD45+ CD56+ | CD16+ activated NK cells |
| 21 | CD45+ CD56+ GrzB+ | Cytolytic NK cells |
| ***Macrophages*** | | |
| 22 | CD68+ HLA-DR+ CD163- | Inflammatory macrophages |
| 23 | CD68+ HLA-DR+ CD163+ | Suppressive macrophages |
| 24 | CD68+ PDL1+ | PDL1-expressing macrophages |
| ***B cells*** | | |
| 25 | CD20+ | Total B cells |
| 26 | CD20+ CD45RO+ | Memory B cells |
| 27 | CD20+ PD-L1+ | PDL1-expressing B cells |
| ***Tumor cells*** | | |
| 28 | PanCK+ PDL1+ | PDL1-expressing Tumor cells |
| ***Tertiary Lymphoid Structures (TLS)*** | | |
| 29 | CD3^hi^, CD20^hi^ immune aggregate associated with PNAd+ venules | Early TLS |
| 30 | Well-defined CD20^hi^ germinal center with extensive CD23 staining or isolated CD23+ FDC staining | Mature TLS |
| ***Eosinophils*** | |  |
| 31 | HALO AI classifier + | Eosinophils |
| CD: Cluster of Differentiation; HLA: Foxp3: Forkhead box protein P3; GrzB: Granzyme B; Human Leukocyte Antigen; HLA-DR: Human Leukocyte Antigen – DR isotype; PanCK: Pan Cytokeratin; PNAd: Peripheral Node Addressin; PD1: Programmed Death-1; PD-L1: Programmed Death-Ligand 1; Tconv: conventional T cells | | |

**Supplementary Table 1. Quantification of select co-expressing cell types, reparative myeloid cell types, and Neighborhood Clustering Analysis for epithelial or combined myeloid clusters.**

|  |
| --- |
| cCR: Complete Clinical Response; NR: No Response; NTL: Non-Treated Lesion; post-tx: Post-treatment; pre-tx: Pre-treatment; TL: Treated Lesion; TLS: Tertiary Lymphoid Structures; Tregs: Regulatory T Cells; TURBT: Transurethral Resection of Bladder Tumor; tx: Treatment. Numbers outlined show cell densities (mean and standard deviation) of early TLS, mature TLS, total TLS, and various cell types and from pre-treatment (pre-tx) and post-treatment (post-tx) TURBT specimens. Samples were further labeled as being from the treated lesion (TL) or non-treated lesions (NTL), and whether a clinical complete response (cCR) or no response (NR) was observed in that lesion. Furthermore, neighborhood clustering analysis was performed percentage areas classified as an epithelial cluster or a combined myeloid, B- and T-cell cluster. p-values were not calculated due to the small cohort (n=5 participants); data is presented in a descriptive fashion. |
